# Current News

**Published:** 1895-10

**Authors:** 


### Current News.

### UNION DENTAL MEETING.

A UNION meeting of the Connecticut Valley Dental Society and the New England Dental Society will be held at Worcester, Mass., October 23, 24, and 25. Many interesting papers and clinics are promised, and an elaborate programme is assured. An important question—Shall these two Societies consolidate, forming a new society, to be called the "Northeastern Dental Association"?—will be decided at this meeting.

GEO. A. MAXFIELD, D.D.S.,
*Secretary Connecticut Valley Dental Society.*
E. O. KINSMAN, D.D.S.,
*Secretary New England Dental Society.*

### DENTAL ASSOCIATION OF NEW SOUTH WALES.

THE Third Annual Meeting was held at the Australia Hotel on Friday, and was well attended. The president, Dr. Burne, occupied the chair, and stated, on behalf of the council, that great hopes were entertained of the Dental Bill being brought before Parliament and becoming law during the ensuing year. The balance-sheet showed a credit balance of seventy-three pounds in hand. It met with general approval. The following officers were elected for the year 1895–96: President, Dr. Burne; Vice-Presidents, Messrs. H. Paterson and S. Chaim; Honorable Treasurer, Dr. W. T. Halstead; Honorable Secretary, Mr. H. Taylor; Committee, Dr. Arthur Hinder, Messrs. C. C. Marshall, F. G. Hollway, J. Darton, H. S. Newton, E. A. Gabriel, and Byron Ruse; Auditors, Messrs. Corbett and Hebblewhite. A vote of thanks was unanimously accorded to the president, who, in thanking the members for their support, referred to the necessity of the profession drawing closer together, and thus securing a higher status. A vote of thanks to the honorable secretary for past services and also to the chairman closed the meeting.

H. Taylor,
*Honorable Secretary.*

---

### SOUTHERN DENTAL ASSOCIATION.

The next annual meeting of the Southern Dental Association will be held in Atlanta, Georgia, commencing the first Tuesday in November. Arrangements are being made for the greatest meeting in the history of the "Southern." The cotton States and International Exposition will be in progress, and railroad rates will be very low. All friends will be given a hearty welcome.

Respectfully,
E. P. Beadles,
*Corresponding Secretary.*

---

### AMERICAN DENTAL SOCIETY OF EUROPE.

The meeting of the American Dental Society of Europe held at Boulogne sur-Mer, France, under the presidency of Dr. Charles W. Jenkins, of Zurich, was one of the most successful and interesting meetings for several years. The next session will be held in Dresden, Germany, in 1896, immediately preceding the meeting of the International Medical Congress in Moscow, thus allowing of attendance upon the two sessions without loss of time.

The officers elected for the ensuing year are as follows:

John H. Spalding, of Paris, President; Charles J. Monk, of Wiesbaden, Vice-President; William A. Spring, of Dresden, Secretary; Samuel S. Macfarlane, of Frankfort, Treasurer.

